# Women's Experiences of Using Anabolic Androgenic Steroids

**DOI:** 10.3389/fspor.2021.656413

**Published:** 2021-11-11

**Authors:** Annica Börjesson, Margaretha Ekebergh, Marja-Liisa Dahl, Lena Ekström, Mikael Lehtihet, Veronica Vicente

**Affiliations:** ^1^Department of Laboratory Medicine, Karolinska Institutet, Stockholm, Sweden; ^2^Division of Clinical Pharmacology, Karolinska University Hospital, Stockholm, Sweden; ^3^Faculty of Caring Science, Work Life and Social Welfare, University of Borås, Borås, Sweden; ^4^Department of Medicine, Huddinge, Karolinska Institutet, Stockholm, Sweden; ^5^Department of Clinical Science and Education, Stockholm South Hospital, Karolinska Institutet, Stockholm, Sweden

**Keywords:** anabolic androgenic steroids, doping, women, phenomenology, reflective lifeworld research

## Abstract

Anabolic androgenic steroids are used by women to increase their muscle mass and because of their performance-enhancing effects. Despite permanent/high risk of side effects, knowledge is inadequate. Our aim has been to deepen understanding about women's use of anabolic androgenic steroids. This phenomenological study is based on the reflective lifeworld research (RLR) approach. Lifeworld interviews were conducted with 12 women, aged 21–56 years, about their experiences of using anabolic steroids. The results show that women experience a sense of pride when they successfully achieve their goals. This is the driving force, triggering tension between suffering and success. Our research adds important knowledge from a reflective lifeworld perspective and shows that women's use of anabolic androgenic steroids is a complex phenomenon. Understanding and knowledge are important in order to be able to meet and support women in their fears and difficulties.

## Introduction

Even though the use of anabolic androgenic steroids (AAS) is considered to be a health problem, little is known about women using AAS, despite the expectedly high risk of side effects, even permanent ones. AAS are categorized as illegal substances in Sweden, but they are effective and can greatly increase strength, muscle, and fat-free mass when combined with strength training (Bhasin et al., 1996; Rogerson et al., 2007). They have also shown a performance-enhancing effect in women (Hirschberg et al., 2020). Typical AAS-induced physical side effects in men include potency problems, acne, and gynaecomastia. Typical psychiatric side effects include depression, sleep disorders and mood disturbances (Sjoqvist et al., 2008). Even though AAS use is mainly a male phenomenon, it is not limited to men. One explanation why women do not use AAS to the same extent may be that women are not as interested as men in becoming very muscular and are more vulnerable to the masculinizing effects of AAS (Kanayama and Pope, 2012) e.g., change of voice, enlargement of clitoris (Strauss et al., 1985; Malarkey et al., 1991). Women generally take fewer substances and lower doses (Borjesson et al., 2016, 2020). In females, sports performance appears to be the main reason for using AAS (Kanayama et al., 2007; Borjesson et al., 2016), especially in bodybuilding and weightlifting (Gruber and Pope, 2000; Phillips et al., 2010). Women identify themselves as competitive bodybuilders or power lifters (Phillips et al., 2010) and appear to be influenced by the men with whom they are in close relationships (Skarberg et al., 2008; Borjesson et al., 2016). Research show that women seek healthcare earlier than men for the negative effects they experience (Garevik et al., 2011; Borjesson et al., 2016).

Currently, no deeper knowledge or understanding exist of women's experiences of using AAS, therefore it is important to study this phenomenon. In this research we turn to the women themselves and to their lifeworld. This is the first study with female AAS users that has practiced the reflective lifeworld research (Dahlberg et al., 2008) (RLR) with a caring science perspective (Dahlberg, 2011). Through the RLR approach, we aimed to reach an existential dimension that is missing in previous research. The results are expected to contribute important knowledge and understanding, especially for healthcare professionals since AAS may affect an individual's health.

## Materials and Methods

### Design

This study has practiced the reflective lifeworld research (RLR) approach described by Dahlberg et al. (2008) and are based on phenomenological epistemology developed by the philosopher Husserl (1970/1936). The study is influenced by a caring science perspective (Dahlberg, 2011) because it is necessary to understand the individual's health in order to be able to support and strengthen the individual in her health process. The lifeworld includes our unique existential world, our experiences and the relationship between them. Through the lifeworld perspective we seek understanding in the lived everyday world. The RLR approach is characterized by an open mind and flexibility toward the phenomenon. The phenomenon being explored and illuminated in this study is women's use of AAS. The researchers needed to have a reflective attitude described as bridling, which involves slowing down the process of understanding and not being too quick to make definite that which is indefinite (Dahlberg and Dahlberg, 2003). To be objective in a phenomenological sense, personal values, theories, and other assumptions may not impede us from acquiring a new understanding of meaning (van Wijngaarden et al., 2017). By not taking anything for granted, we can take control over our preconceptions.

### Participants and Study Setting

A total of 12 women participated voluntarily in the study. All were current or former users of AAS. They were recruited in two ways: either via snowball sampling or when contacting the Anti-Doping Hot-Line. The participants had to be over 18 years of age, as this is the age of majority in Sweden, and their age range was 21–56. Participants were required to understand the Swedish language and were included in the study in the order they came in contact with the interviewer. Snowball sampling (Heckathorn, 2011) is a convenient method to get in touch and generate informants in hard-to-reach populations. It involves an initial contact, in this case AAS users, who in turn can generate new informants. The Anti-Doping Hot-Line has been organized since 1993 as an anonymous free telephone counseling service for people concerned about or affected by their non-medical use of AAS (Eklof et al., 2003). The Anti-Doping Hot-Line started after observations of the need in society for an information service about the health risks of doping. The service is managed by trained nurses and clinical pharmacologists at the Department of Clinical Pharmacology, Karolinska University Hospital Stockholm, Sweden.

### Data Collection

The interviews lasted between 45–90 min and were tape-recorded and thereafter transcribed verbatim. To maintain privacy, the interviews took place upon the informant's request in a separate room at a library or in the informant's home. One of the interviews took place in a café based on the informant's request. Each interview started with a presentation of the study's aim and then continued with the main open question: “How is it to use anabolic androgenic steroids?” Follow-up questions were asked (e.g., how do you mean, can you describe more?) to capture the individual's perception and to gain deeper insight into the phenomenon.

### Data Analysis

The analysis followed the phenomenological approach in accordance with the guidelines for RLR (Dahlberg et al., 2008). Analyzing data according to RLR is about understanding the phenomenon and finding its meaning, and abstraction is carried out by referring back and forth between the whole and its parts, and then reconstructing the whole. This is a process to understand different abstract levels of meaning when seeking for the essence of the phenomenon. The focus on the phenomenon is of crucial importance in this process. The informants had described their experiences in the interviews, which means that they had delivered data to be analyzed. A rich material in meanings and variations of these meanings is necessary to be able to reach the essential description of the phenomenon. In the analysis process, however, we concentrate on the phenomenon, which means that the analysis is not subject-orientated, but phenomenon-orientated. In other words, we seek for meanings in the experiences collected, that constitute the phenomenon. The essence presents the meanings of the phenomenon on an abstract and general level. Thus, the total result of the study describes the phenomenon in a general sense, which is not solely related to the informants in the study. In order to find the essential meaning of the phenomenon, the analysis work must be carried out with a reflective and bridling attitude according to the RLR approach (Dahlberg et al., 2008). According to Brinkmann and Kvale (2014), the data must be divided, organized, and simplified to get a clear picture. The analysis began with listening and reading the interviews in their entirety with an open mind to facilitate an initial understanding. After repeated reading, the transcript was divided into meaning units to search for meanings. From a phenomenological perspective and validity research should be meaning-oriented (van Wijngaarden et al., 2017). When the interviews were emptied of all meaning, the meanings were clustered together to find similarities and differences. A pattern of meanings slowly emerged and shaped a meaningful structure that constitutes the essence of the phenomenon. The aim is then to describe the variations and nuances of the phenomenon, which means the constituents. This means that the focus is still on the phenomenon, but the nuances are illustrated with quotes from the informants.

### Ethics

Ethical approval was obtained from the Regional Ethics Committee at the Karolinska Institutet, Stockholm (nr. 2016/1762-31/5). Before and on the occasion for the interview, the informants received oral and written information about the purpose of the study, stating that participation was voluntary and confirming their right to withdraw if they so wished, without explanation. Information was also given about the confidentiality of the interviews. Written consent was given by the participants. After the interviews, the participants in the study were offered care and support if needed.

## Results

The results are first presented by the essential structure of meanings, followed by its five constituents.

Living with AAS can be hard in many ways and difficult to endure. It involves existential challenges to achieve the perfect body. Body dissatisfaction creates anxiety, which is mastered by hard training, strict diet and the use of AAS. The ambition is to use training, diet, and AAS as the means to acquire a perfect body as well as recognition and social acceptance. The experience of succeeding through their achievements creates a sense of pride, which is the driving force, triggering tension between suffering and success. Lack of self-esteem contributes to the experience of the body's imperfection. Low self-esteem is compensated for by self-control, discipline and performance. Using AAS means living with feelings of fear, guilt, shame, and vulnerability. It is an arduous endeavor to balance the substances' side effects with desired femininity. Existing standards of femininity casts a permanent shadow over existence. Constant search for knowledge leads to insights about the use of AAS. A self-preoccupation is shown as emotional coldness toward and distanced from people around. The use of AAS also means living with lies and the fear of being discovered, because AAS are illegal.

### Striving for the Perfect Body

In striving for the perfect body, women live with body anxiety, which means experiencing that their bodies are not perfect. To manage this anxiety, they begin strength training in order to build muscles. This allows them to eat more without gaining weight. Eating disorders, previously part of their lives, have made them aware that exercise helps to avoid the problem of weight gain.

One woman recalled:

“*The first times? Well, I had bulimia and so on before…that was perhaps also part of the reason for starting this training thing, and also generally that I realized that I could then eat without having to puke. Instead I had to eat to gain muscle, and then it became like periods of building up when it was okay to eat and exercise hard and I didn't put on weight because I gained muscle”*.

Despite hard training and the use of AAS, women may still feel that they are not achieving what they want. The feeling of dissatisfaction persists even though their muscles are getting bigger. Their distorted body image makes it difficult for them to perceive their own bodily changes realistically and also to receive positive comments from others. To gauge their progress, they ask selected people for advice, look at photographs of themselves, use tape measures or try on clothes. Even when they perform well, feelings persist of their results not being good enough, and they start to focus on the next set or new goals.

One woman described her feeling of dissatisfaction with her bodily changes:

“*We were all influenced by our idols, and how they looked on stage. We read magazines to see ourselves in that role or in that situation and perhaps someone felt that her shoulders weren't good enough. If her shoulders were okay, then perhaps her legs were wrong. All the time, our aim was the perfect physique with all the muscles in harmony with each other. Genetically, everybody has a muscle group or a body part that genetically doesn't develop as quickly”*.

A strictly controlled diet is helping the women in their achievements of reaching the perfect body. Scheduled eating days are planned when eating more freely is permitted, i.e., food that is not normally allowed. It is easy to eat more than planned and sometimes food intake goes out of control. A feeling then arises of bodily collapse, creating body anxiety and resulting in compensatory training. This can be particularly difficult in periods, especially after bodybuilding competitions. Therefore, these women constantly continue with the same strict diet. Prior to competitions, small changes in diet can be perceived as crucial, as one woman noted:

“*I came second in the Swedish championship and I had cheated by eating 4 grapes two weeks before the competition. It wasn't really cheating because everyone else did it too, but I felt it was cheating and I came second in that competition. In other words, could I have won if I hadn't eaten those grapes? is going round in my head. Before, I couldn't even use lip balm because it contained fat and I was scared of getting it into my body. I was so scared of everything that could sabotage a diet or a commitment, because it meant my whole life to me”*.

Before women start using AAS, they need to have a basic physique. When this stage is reached and the body can no longer develop naturally, they feel that a careful use of AAS is justified.

### Increasing Self-Esteem Through Performance

Women control their lack of self-esteem through their performance. It is important to be successful to counteract early fears of not being good enough. A life-history with eating disorders, bullying, negative comments about appearance, lack of recognition, and lack of love is common.

Lack of self-esteem is regulated through self-control and discipline and this is achieved mainly by following strict dietary and hard exercise routines. Building one's body provides the opportunity to demonstrate skills and value. People recognize and look up to bodybuilders as individuals, making them feel successful and strengthening their experience of being determined, disciplined and healthy.

One woman reflected:

“*I think many people come from a very destructive background, so many have pushed themselves hard before with eating disorders or other destructive things. Because it's not really healthy to push yourself so hard…so you have to be hard-headed and that comes from somewhere. Either your upbringing was tough, or you're prepared to fight even though it hurts. I don't know, perhaps pushing yourself or punishing yourself makes it clearer in some way. Because afterwards you get rewarded a little by time in the limelight and attention for all your hard work”*.

Physical development thus leads to approval and attention from other people. This increases personal status and motivates further and better performance. Living healthily creates a feeling of superiority to others. However, there are also thoughts that the result has not come quite naturally.

### Maintaining One's Femininity

To avoid masculinising side effects and over-large muscles, the intake of AAS needs to be balanced. Women are uncertain about being able to handle this balancing act and live in a fear of losing their femininity. They have an inner limit for acceptable side effects, so they struggle to maintain the balance between desirable muscle development and acceptable side effects. Not being able to get pregnant, and permanent side effects such as clitoral enlargement, increased body hair or a deeper voice frighten them. However, in order to develop in training and to have a realistic chance of meeting other people in the bodybuilding sport, certain risks must be taken. If muscle development is too slow, thoughts may appear of increasing the dose or switching to a more potent substance. However, if side effects occur, the dose may be reduced or discontinued.

One woman commented:

“*But nothing happened. So then I thought, well, I can just keep going. Every time I had the injection, I felt such anxiety in my body. I repeatedly checked my clitoris, I searched on Google and read about clitoris enlargement about 100 times”*.

Knowledge about AAS and how to use the substances is required in order to hide the use of AAS from others. Being well-informed and critical of one's sources reduces the risk of both side effects and being deceived into making the wrong choices. The concern about and fear of incorrect advice and the authenticity of the substances make it impossible to trust advices from others. The women are usually led by men who give them advice based on how men use AAS. They request first-hand information about how AAS works in a female body but rarely exchange experiences with each another. The women think that societal information from a female perspective where not only the negative effects are described would increase credibility. People's views on femininity are affected by traditions and societal norms in terms of appearance and appropriate clothing. Women with large muscles are questioned by others.

One woman described her own experience of not fitting in:

“*When my body got muscles, they roared with laughter at me and said that the men's department is on the other side of the street…you feel a bit….divided about how to dress yourself if you're very muscular. If I go in dresses or skirts and stuff, then I feel like people are looking at me like I'm a transvestite. For a while I thought it was really hard because….it was like this, I was almost…I don't know…not hurt but I thought it was hard to constantly have to stand up for myself and I had to fight all the time to….do I need to prove that I am a girl or what….or do I look like a guy”*.

A muscular appearance makes women vulnerable. Disgusting comments and shameful suggestions to women with muscular looks come from men in social media who are fascinated by women with strong, muscular bodies. Unwanted confirmation in daily life occurs too: unknown people pinch and feel women's bodies without warning and without asking for permission. One woman described her experience of comments in social media:

“*Is it okay to behave like this, what if someone had been fat – why are you so fucking fat, is it okay to say things like that? – and besides, I have a fan page on facebook, a page where you go to look at pictures of me, why the hell should they go there and look at pictures of me and then make negative comments? They should shut up and scroll on if they don't like a picture”*.

### Self-Preoccupation Impedes Social Life

These women need to focus to be able to attain their goals, and this need tends to exclude family and friends. Their self-centered behavior mainly revolves round routines related to food and exercise. A bodybuilder's lifestyle is tough and time-consuming. In addition to foodplanning and several workouts per day, most are employed and have to work. It is important to get enough sleep, and time for the family's activities as well as socializing is thus limited. One driving force is being able to show themselves and their families that they can succeed and that sacrifices have not been in vain. Having one's own experience and understanding of what it means to disappear into the “bubble” where only exercise and food exist also makes it easier to live with a bodybuilder.

One woman related what sometimes took place:

“*…uh but we can do this tomorrow instead, let's go to the cinema tomorrow, I'm too tired today. So we plan to go tomorrow and then didn't make it…or you'd like to go to the park and then you feel too tired, maybe we can watch TV instead. So it was like, early mornings with the children when they were very small, a 1-year-old and a 2-year-old, it was winter and I'd go out on my morning walk with a twin stroller, putting two children in a stroller each with a comic at 4 in the morning and then going out and ploughing through the snow like. It's not much fun for two small children…”*

Another woman told a different story:

“*I didn't care very much about what happened to my ex and that the children chose to move. It became less important and didn't bother me that much anymore. I started to sleep well at night, and I was only living for my workouts and substances. In a way, it was like going into a “bubble” and completely ignoring what was happening outside the “bubble”. Of course, I was a selfish person, 100 % selfish because I only thought of myself and maybe even ignored how the children were doing. I thought it was more important to exercise than have time with my children, it was scary that it became so emotionless”*.

When empathy is lacking, conflicts, and/or an agitated mood can easily result. Sometimes, people get in the way of performance, and it is usually planned routines that are disturbed by others e.g., when exercising. Relationships may be perceived as demanding under stressful conditions. Daily routines such as controlling one's body can be time-consuming when one is preoccupied with oneself.

One woman compared her past with AAS and the present:

“*In other words, nowadays I feel that I only need to look at myself in the mirror before leaving in the morning and then again when I get home. Nowadays, I might examine my face for specks during the day, but at that time it was totally insane. I mean, before that time, I've never ever liked to see myself naked or in lingerie, but then I almost had to, I wanted to take off most of my clothes so I could see every part of my body. I studied my body for about one hour every day, which is completely insane. And I liked what I saw, it was a strange feeling to love looking at yourself. Narcissus in other words, it was like a moment of love every day to be able to see myself naked”*.

Traveling, education, and time with the family get excluded when the focus is on bodybuilding. This conscious sacrifice may make these women wonder if it is worth missing “normal life.” However, it may be difficult to create a new identity with less focus on muscles and to stop admiring what one has looked up to for so many years.

### Living With Lies

The secret use of AAS requires women to live with lies. The fear of this secret use being revealed is constant, if the physical changes and side effects were to be noticed by others and lead to social consequences and penalties.

One woman admitted her concerns:

“*I actually had nightmares that the Police would take me and my children would be alone. Then I stopped at once. That fear was horrible. But after a couple of years, I started again”*.

Women often ignore any side effects and hope that others will not notice them either. They try to protect themselves by hiding their physical development and disguising visible and invisible side effects e.g., they hide their bodies in larger clothes and/or try to avoid doping tests.

The use of AAS is not only illegal but also charged with taboo and must therefore take place in secret to reduce the risk of being exposed. Honesty is not possible when using AAS, because society is judgemental and condemns the use of AAS as cheating. Therefore, certain social situations have to be avoided. It is important to keep one's self-image as a good person untarnished. Being a good person means being physically fit and well-trained, disciplined and healthy, and not being a person who uses forbidden or illegal substances or has an “artificially built body.” Exposure for using illegal or forbidden substances can generate severe feelings of shame. Lying is a tool to escape shame and avoid being confronted by disapproval and rejection. Only a few people, among the closest and most trusted, know about a person's use of AAS.

One woman confessed:

“*After all, I was a personal trainer and role model for many people who admired my physique. I was a role model for them, and I know that everything would have collapsed if it had become widely known that I have injections regularly to look like I do”*.

Women sometimes withdraw to avoid questioning and non-accepting people who do not share the same values. For example, they hide with their lunch box in the toilet to follow their special diet in secret. The choice to spend time with people who share the same lifestyle is made easier by friends who do not understand the need to take one's own food to a party or decline to participate in activities because of the need to exercise.

One woman listed some of the questions she hears from other people:

“*Can't you just eat normally?” or “Are you always going to do that?” or “Are you going to live like that for the rest of your life?, haven't you got a life?, aren't you going to live normally?, are you going to keep on like this forever?”*.

## Discussion

The women described perfectionist traits showing their expectations of achieving the perfect body. They were hard on themselves, driven by unhealthy ideals, sought confirmation, were self-critical and constantly saw their bodies' faults and shortcomings. Perfectionist behavior often involves excessively high demands (overcompensation, excessive control, correction) and the pursuit of flawlessness. These characteristics are also typical for elite athletes (Lemyre et al., 2008). According to Stoltz and Ashby (2007), there is a satisfaction and nothing wrong in trying to reach perfection (adaptive perfectionism). It is when a healthy striving gives way to self-imposed demands, self-critical evaluations of achievements and concerns about negative assessments (maladaptive perfectionism) that it can become unhealthy. The unhealthy part is not being able to accept oneself unless one is perfect, and constantly considering that one could do better (Lundh, 2004). Perfectionism has increased in recent decades. This may be due to individualistic and materialistic environments with greater competition, higher demands, and unrealistic expectations (Curran and Hill, 2019). International valuation surveys show that Sweden ranks high on the scale of values for individualism and self-expression (World Values Survey, 2015). Individuals in the Western world are positively committed to increasing their physical strength and to setting high goals (Lo et al., 2011).

In our research, the women described that achievement was important to them. The women had experiences of not feeling loved, not being good enough or not receiving recognition. Their concern for and fear of not peaking in performance made them strive even more and even harder. They needed to show others and themselves that they were self-disciplined women. However, they never felt that they attained their goal even though they looked perfect to others. This feeling of non-attainment caused lack of self-esteem. Performance-based self-esteem (PBSE) (Hallsten et al., 2005) is a variant of low self-esteem that is based on the individual proving her human value through performance. It is based on a concern for not being good enough and the results achieved are an important measure of ability (Hallsten et al., 2005; Makower, 2018). PBSE indicates how self-esteem is created and how it is maintained, and is a concept linked to perfectionism (Hallsten et al., 2005). Individuals with high PBSE are often ambitious and base their value on external factors such as success and personal status. External confirmation becomes a compensation for their lack of self-esteem (Hallsten et al., 2005). Chasing achievements and positive feed-back from other people may lead to negative consequences such as stress and exhaustion (Svedberg et al., 2016). Individual characteristics as well as inadequate support in working life and from family have been showed to trigger PBSE (Blom, 2012).

The results show body dissatisfaction and strong focus on increasing muscle mass in the individuals interviewed. Body anxiety occurred when routines around exercise or diet were disturbed. Muscle dysmorphia (MD) is a form of body image disorder characterized by a preoccupation with muscularity and body image (Phillips et al., 2010). Individuals with MD describe a dissatisfaction with their bodies and a desire to be more muscular. Male bodybuilders report a greater incidence of MD where the focus is on strict diet, extremely heavy weight training and the use of AAS (Pope et al., 1997; Mitchell et al., 2017). Female bodybuilders probably tend to have the same risk of developing MD as men (Hale et al., 2013) but have been investigated to a very small extent (Gruber and Pope, 2000). Men with MD experience symptoms of anxiety when exposed to environments where the body can be seen (Olivardia et al., 2000).

Women live with lies because the use of AAS is forbidden and illegal and occurs in secrecy. Its exposure would therefore feel shameful. Also, people generally are unable to understand the sacrifices needed to reach the goals of bodybuilding. They hid their bodies or avoided certain social situations. In MD hiding a body are described as a part of the symptomatology (Pope et al., 1997) to not experience anxiety. This kind of anxiety was not seen in our results. Our view, however, leans toward that women are concealing their bodies due to fear of being exposed for illegal activity rather than fear of being judged for their appearance.

Developing muscles requires adequate, monitored food intake. Eating was itself a major reason why women started with weight training. A previous eating disorder made it easier for them to handle a strict diet. Diet increased their control over their bodies and reduced their anxiety. Unlike their previous experiences, eating became justified. Eating disorders have been described in male bodybuilders with MD (Pope et al., 1993; Mitchell et al., 2017), in female bodybuilders (Gruber and Pope, 2000; Phillips et al., 2010), in maladaptive perfectionism (Dahlenburg et al., 2019), and in athletes in weight control sports (Thompson and Sherman, 2014). Despite this, the fear existed of not eating right or being able to handle a controlled diet, as has been described previously in female bodybuilders (Gruber and Pope, 2000).

The women in this study were trying to create a balance for themselves with regard to their physical attributes, somewhere in the border area between what is considered masculine and what is considered feminine. Their distorted body perception made it difficult for them to apprehend how muscular they were. It is not easy to know the limit for side effects and when they will occur, a fear exists of being masculinised (Sverkersson et al., 2020). The advantages and disadvantages of AAS regarding masculinisation need to be weighed against each other and evaluated. This must be done because women are more vulnerable than men to the negative effects of AAS and are more susceptible to side effects (Strauss et al., 1985; Gruber and Pope, 2000). It was important for the women in this study to maintain their femininity and regulate the size of their muscles. Strong, muscular women are not perceived as feminine and are not an accepted norm in society. According to the social constructivist perspective, we are born into a society that constantly influences us where we relate to existing norms and conceptual frameworks (Goffman, 1959). We are brought up in different gender roles, for example, how we should dress, look and behave. Traditionally, the hegemonic understanding of the female body is weak and fragile in contrast to men's which is both big and strong. In addition, women's appearance should also appear natural (Goffman, 1977). When people are of the same opinion, a general perception is formed that leads to the creation of ideals. Body perception is influenced by the appearance ideals that exist in society (Goffman, 1959). Bodybuilding women have the potential to both accept and subvert the ideal (Tajrobehkar, 2016).

The results showed that women were concerned and had a fear of getting incorrect advice from others and being deceived into making the wrong choices. They seldom exchanged experiences with each another and were led by men who gave them advice on how to use AAS. The women only trusted their own knowledge and had a critical approach to information. The women's networks are smaller and newer when it comes to AAS, but it has been shown that they follow a similar path for acquiring knowledge as men (Henning and Andreasson, 2019). The discussions at the online forums, where women are also present, are usually dominated by men who share their experiences and give advice. Women prefer first-hand experiences from other women (Sverkersson et al., 2020) and not advice from men, this makes it more difficult to sort among information (Henning and Andreasson, 2019). Online communities only for women are though emerging (Andreasson and Henning, 2021), which enables for women to discuss their use of AAS from a female perspective (Sverkersson et al., 2020). There is a need for credible societal information from several perspectives, i.e., not only the negative aspects. It is also important to take women's experiences into account when designing targeted prevention strategies, especially when it comes to the fear of exposing their use.

Women with muscles challenge what is considered to be the sphere of men (Sverkersson et al., 2020). The women in this study are potential role models who, through their practice, go against the hegemonic view of femininity and norms of how women should be. They crush the notion of femininity as something fragile (Tajrobehkar, 2016) and possess a strength with their efforts to change their bodies, beyond stereotypical gender configurations (Sverkersson et al., 2020).

There are not many women using AAS and it is a hard-to-reach population. A total of 12 interviews were included in this study. This may be seen as too few to allow any conclusions to be drawn or to be representative of all women using AAS (Polit and Beck, 2017). However, through the use of a phenomenological research approach with a lifeworld perspective, the data collection has contributed with meanings forming the basis for an analysis and generating an essential description of the phenomenon: women's use of AAS. The results thus constitute knowledge development for understanding women in general who use AAS.

The informants gave their descriptions based on their lived experiences. However, since the intake of AAS is illegal under Swedish law, there is an increased risk that the informants may have chosen their answers to protect themselves. One of the interviews took place in an undisturbed part of a café, this may also have affected the informant's way of answering. However, with the help of in-depth interviews and support for reflection, substantive meanings have emerged. In this way we believe that the material is truthful. In-depth interviews as a method have been shown to be a strength in order to gain a deeper understanding. The method is time-consuming which is a weakness and requires knowledge in interview technology.

## Conclusions and Clinical Implications

The overall knowledge in society about women's use of AAS is very low. Men's use of AAS is known to healthcare, but no deeper understanding or knowledge exist of the phenomenon in women. Women's use of AAS is a complex phenomenon. The results show that women experience a sense of pride when they are successful in their achievements. This is their driving force, triggering tension between suffering and success.

Understanding and knowledge are important in order to be able to meet women in their fears and difficulties. This research contributes knowledge that provides support to the development and improvement of prevention and treatment strategies, not only medically but also psychologically and socially. Gender needs to be considered when disseminating information. The law in Sweden imposes barriers since use is illegal. Because of this, anonymous telephone counseling is a good solution for providing support and advice.

## Data Availability Statement

The original contributions presented in the study are included in the article/supplementary material, further inquiries can be directed to the corresponding author/s.

## Ethics Statement

The studies involving human participants were reviewed and approved by the ethical approval was obtained from the Regional Ethics Committee at the Karolinska Institutet, Stockholm (nr. 2016/1762-31/5). The patients/participants provided their written informed consent to participate in this study.

## Author Contributions

AB and VV designed the study. The interviews were carried out and transcribed by AB. All the authors have read and approved the final manuscript, and analyzed the data.

## Conflict of Interest

The authors declare that the research was conducted in the absence of any commercial or financial relationships that could be construed as a potential conflict of interest.

## Publisher's Note

All claims expressed in this article are solely those of the authors and do not necessarily represent those of their affiliated organizations, or those of the publisher, the editors and the reviewers. Any product that may be evaluated in this article, or claim that may be made by its manufacturer, is not guaranteed or endorsed by the publisher.
